# Interface Fluid Syndrome Masquerading as Diffuse Lamellar Keratitis After Small Incision Lenticule Extraction

**DOI:** 10.7759/cureus.36832

**Published:** 2023-03-28

**Authors:** Stephen LoBue, Kelli Coleman, Peter Lam, Christopher Shelby, Wyche T Coleman

**Affiliations:** 1 Department of Ophthalmology, Willis-Knighton, Shreveport, USA; 2 Department of Ophthalmology, Louisiana State University Health Sciences Center, Shreveport, USA

**Keywords:** small incision lenticule extraction (smile), steroid-induced ocular hypertension, diffuse lamellar keratitis (dlk), pressure-induced stromal keratopathy (pisk), interface fluid syndrome (ifs)

## Abstract

A 34-year-old male with no past medical or ocular history underwent bilateral uncomplicated small incision lenticule extraction (SMILE). On day 1, uncorrected distance visual acuity (UDVA) was 20/25 in the right eye (OD) and 20/20 in the left eye (OS). The intraocular pressure (IOP) was 12 mmHg in both eyes (OU). On day 17, UDVA was 20/70 OD and 20/30+2 OS. Slit-lamp examination (SLE) revealed diffuse 2+ haze at the interface suspicious for diffuse lamellar keratitis (DLK). Topical difluprednate was added twice a day (BID). Vision decreased by day 20 with a significant myopic shift and 3+ interface haze OU. A washout of the interface was performed. Topical steroids were increased with oral prednisone. One day after the washout, vision and interface haze improved. On day 3 status post washout, UDVA decreased to 20/70 OD and 20/50 OS. IOP was 42 mmHg OU. A diagnosis of interface fluid syndrome (IFS) was confirmed. All steroids were stopped while adding ocular hypotensive medication. One month later, visual acuity was 20/20 OU with a complete resolution of interface haze.

Only a handful of IFS has been documented in SMILE, an incidence that may increase as SMILE becomes more common. Among all SMILE cases, IFS was most commonly associated with steroid-induced ocular hypertension and a myopic shift around 21 days postoperatively. A fluid cleft at the interface may not always be visible with SLE, masquerading as DLK. Scheimpflug densitometry and anterior segment optical coherence tomography (AS-OCT) may aid in quantifying interface edema needed to confirm a diagnosis when IOP is unclear. A corneal washout can immediately improve corneal edema, but the preferred treatment is discontinuing all steroid medication and starting glaucoma drops.

## Introduction

Small incision lenticule extraction (SMILE) is a relatively new refractive procedure that involves a small superior incision used to remove a central button of corneal tissue known as the lenticule.

Although SMILE avoids the flap-related complications associated with laser-assisted in situ keratomileusis (LASIK), the procedure is technically more challenging. Varying intraoperative complications specific to SMILE are possible, including difficulty in lenticular dissection or extraction, cap perforation, and decentered centration [1]. Various postoperative problems have been documented, including interface debris, epithelial ingrowth, infectious keratitis, diffuse lamellar keratitis (DLK), and transient light sensitivity syndrome (TLSS) [2].

Alternative rare complications of both SMILE and LASIK include similar terminologies of pressure-induced stromal keratopathy or keratitis (PISK) or interface fluid syndrome (IFS). Both have similar, if not identical, presentation with fluid retention within a potential space of the cornea including a LASIK flap or lenticular cap after SMILE.

However, the mechanism of corneal edema differs as PISK, by definition, requires elevated intraocular pressure, while IFS includes alternative causes of endothelial dysfunction. As a result, IFS has become a more inclusive term to describe a wider variety of pathology depicting a similar clinical presentation. Thus, we will use the term IFS for the remainder of this case.

IFS, although uncommon, has been more widely documented in post-LASIK patients. Only four previous cases in the literature have documented IFS in post-SMILE patients, despite over seven million procedures being performed worldwide.

To better understand the clinical presentation, time of onset, and diagnosis techniques of IFS in post-SMILE patients, we present the fifth reported case and review all documented cases in the literature.

## Case presentation

**Figure 1:**
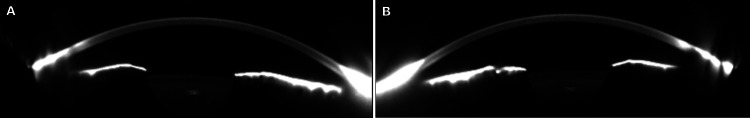
Normal Anterior Segment Scheimpflug images of the anterior chamber, highlighting a clear cornea with no opacities of the (A) right eye and (B) left eye.

A 34-year-old male with no past medical or ocular history presented to the Willis-Knighton Eye Institute to undergo evaluation for refractive surgery. The patient had a best-corrected visual acuity (BCVA) of 20/15 in both eyes (OU) with a manifest refraction of -8.75 in the right eye (OD) and -8.50 in the left eye (OS). Slit-lamp examination (SLE) demonstrated a clear cornea without opacities (Figure 1).

**Figure 2:**
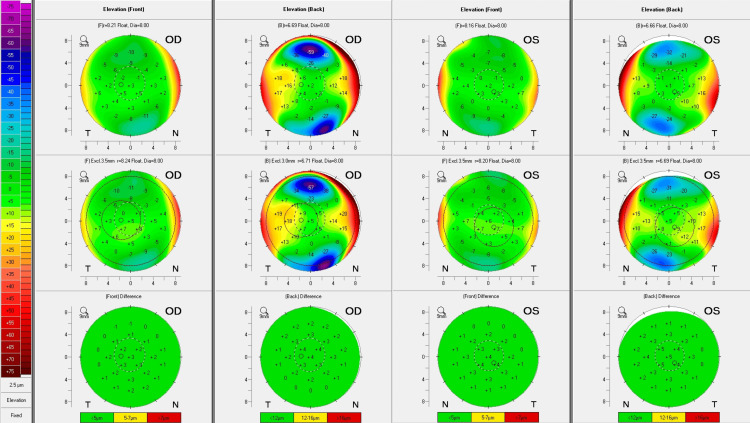
Corneal Tomography Corneal tomography demonstrating normal levels of anterior and posterior corneal elevation in the OD and OS. No signs of underlying ectasia risks were observed in either eye. OD: right eye, OS: left eye

The anterior chamber was deep without signs of inflammation OU. The intraocular pressure (IOP) was 12 mmHg OD and 14 mmHg OS with a central corneal thickness (CCT) of 552 OD and 538 OS. The fundus examination was unremarkable with a normal optic nerve, macula, and peripheral retina. The patient was at a low ectasia risk with a low D score of 0.69 OD and 0.59 OS. Kmax was also low at 43.4D OD and 43.0D OS. The Belin/Ambrósio enhanced ectasia display demonstrated normal levels of anterior and posterior corneal elevation OU. No signs of underlying ectasia risks were observed in either eye (Figure 2).

The patient underwent a bilateral uncomplicated SMILE procedure. The procedure was uncomplicated with complete lenticule extraction and visualization. On postoperative day 1, uncorrected distance visual acuity (UDVA) was 20/25 OD and 20/20 OS. The IOP was 12 mmHg OU with applanation tonometry. A well-healing superior corneal incision and a clear cap were noted without anterior chamber inflammation. The patient was started on preservative-free artificial tears four times a day (QID), gatifloxacin 0.5% QID, and prednisolone acetate 1% QID.

However, on postoperative day 17, the patient returned to the clinic complaining of worsening distance vision with difficulty driving. UDVA was 20/70 OD and 20/30+2 OS. IOP was not taken at this time. SLE revealed 1+ haze at the interface with signs suspicious for DLK. Prednisolone acetate 1% was discontinued, and topical difluprednate was added twice daily (BID).

**Figure 3:**
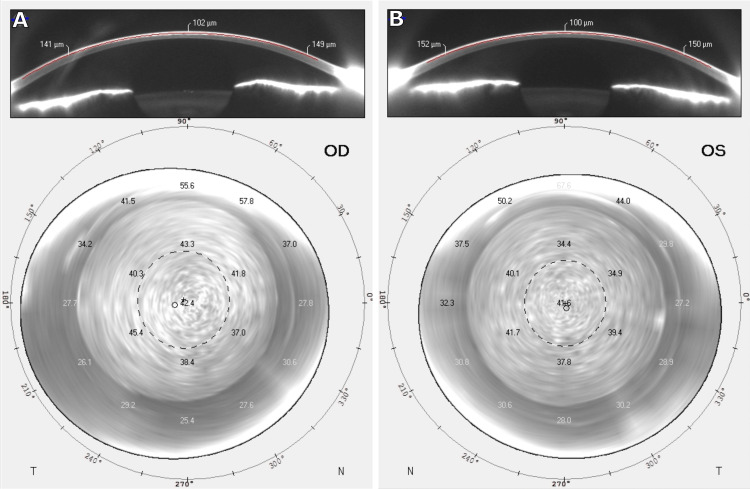
Automated Scheimpflug Densitometry Automated Scheimpflug densitometry demonstrating a dense haze within the cap with corneal edema in the (A) right eye and (B) left eye. Central corneal thickness was (A) 488 μm and (B) 471 μm. OD: right eye, OS: left eye

The patient returned on postoperative day 20 with complaints of worsening distance vision. UDVA was 20/400 OD and 20/150 OS. BCVA was 20/50 OD and 20/30 OS with a refraction of -4.25 sphere and -2.75 sphere, respectively. SLE revealed significant interface haze 3+ bilaterally. Pentacam (Oculus Wetzlar, Germany) Scheimpflug densitometry demonstrated a dense haze within the cap (Figure 3).

The patient was evaluated by another experienced refractive surgeon on the same day. Worsening DLK was suspected. A washout of the interface was performed, and cultures were obtained. The patient was started on prednisone PO 60 mg daily, and polymyxin B/trimethoprim was added QID. The patient was instructed to continue difluprednate six times daily and gatifloxacin QID.

**Figure 4:**
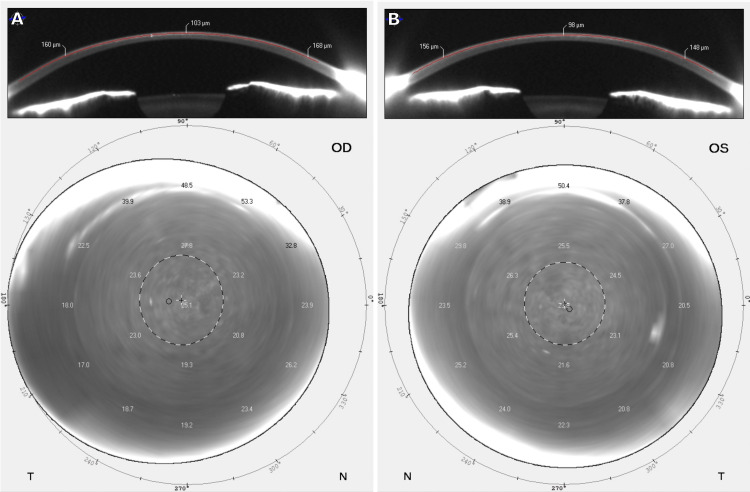
Automated Scheimpflug Densitometry Automated Scheimpflug densitometry demonstrating improvement in corneal haze and edema within the cap in the (A) right eye and (B) left eye. Central corneal thickness was (A) 447 μm and (B) 444 μm. OD: right eye, OS: left eye

One day after the corneal washout, the patient returned reporting an improvement in vision with minimal pain. UDVA was 20/50-2 OD and 20/40+1 OS. IOP was not performed. On SLE, there was mild inflammation at the interface bilaterally with minimal haze. Pentacam Scheimpflug densitometry demonstrated improvement in corneal haze within the cap (Figure 4).

The patient was instructed to continue current medications and return in two days.

Day 3 status post washout, the patient presented with worsening UDVA of 20/70 OD and 20/50 OS. Intraocular pressure with peripheral applanation was 42 mmHg OU. A diagnosis of IFS was made at this time. All steroids were stopped. The following medication was started: brimonidine/timolol BID, bimatoprost daily, and acetazolamide 500 mg PO BID.

Day 4 status post washout, the patient’s visual acuity improved to 20/50 OD and 20/40 OS. IOP were noted to be 8 mmHg OD and 7 mmHg OS on rebound tonometry. At one-month status post washout, the visual acuity was 20/20 bilaterally. His only complaint was mild dysphotopsias at night. IOP-lowering medications were slowly decreased over three months, and visual acuity improved to 20/15 bilaterally at nine months. The patient was very pleased with his result off all medications and topical drops.

## Discussion

Although the occurrence of IFS after LASIK has been well described, only a handful of cases pertaining to SMILE have been reported [3-5]. After performing a thorough literature review, we present the fifth reported case of IFS post-SMILE in the literature [6-9]. Thus, we have compiled a case report and review to better understand the clinical presentation, time of onset, and diagnosis of IFS in post-SMILE patients.

As with our case, IFS has been known to be mistaken for DLK after refractive surgery [3]. According to Moshirfar et al. [1], a classic diagnostic error among clinicians involves misinterpretation of interface scatter as DLK.

DLK typically presents in the first postoperative week with symptoms of pain, photophobia, redness, or tearing with a nonlocalized, subepithelial corneal haze confined to the flap [10]. Although IFS typically has a later onset, DLK has been reported months after LASIK [11,12].

Similar to DLK, IFS can have diffuse interface haze. Fluid at the interface wound is variable and may not be visible at the slit lamp [13]. A grading scale from 1 to 3 for IFS has been described [14]. IFS stage 2 can simulate DLK stages 1-2 [14]. Although elevated IOP is a prominent feature of most IFS cases, IOP may be artificially low secondary to the fluid interface, especially with Goldmann applanation tonometry (GAT). Noncontact or handheld electronic tonometry has documented improved accuracy in cases of corneal edema [15,16]. However, if not available, peripheral GAT may allow for improved IOP measurements.

Although our case of IFS may have been obvious in hindsight, the diagnosis was not clear to us in the initial presentation. The physical findings were consistent with previously observed post-LASIK DLK, and our lack of consistent IOP measurements with rebound tonometry delayed our diagnosis. At the time of this case, we did not have rebound tonometry and did not routinely check IOP on refractive surgery patients in the immediate postoperative period. We were not alone in this practice as many were hesitant to use GAT, especially among technicians, in fear of causing mechanical flap or corneal trauma with applanation. However, with the advent of rebound tonometry, we believe that IOP can be safely checked in the immediate postoperative period with minimal risk to the corneal flap or cap in the case of SMILE.

Clinically, our patient presented with diffuse corneal haze at postoperative day 17, which worsened by day 20 after starting topical difluprednate. The patient had a significant myopic shift in refraction with corneal haze and edema seen on automated Scheimpflug densitometry. Consultation with an additional refractive surgeon in person, as well as two others via phone, led to a strong suspicion of DLK with the recommendation of a washout. Vision, interface haze, and edema significantly improved after the washout, leading us to believe that DLK was the etiology. However, reevaluation of the patient on day 3 status post washout demonstrated worsening vision with an IOP of 42 mmHg OU with peripheral GAT. In hindsight, the initial improvement from the corneal washout was due to the removal of excess fluid within the cap confirmed by a decrease in CCT. Topical glaucoma drops were started with PO acetazolamide. Topical and PO steroids were stopped. IOP-lowering medications were very slowly decreased over three months, and visual acuity improved to 20/15 bilaterally at nine months.

Reviewing all cases of IFS after SMILE revealed a mean onset of 17.2 ± 8.3 days (mean ± standard deviation). Decreased VA with an interface haze appeared in all cases with a significant myopic shift secondary to corneal edema in 80% of cases (Table 1). Excluding the case that had an intraoperative complication of a cap tear, the remaining uncomplicated SMILE cases developed IFS secondary to elevated IOP (Table 1) [8]. In all these cases, elevated IOP was secondary to a steroid response around 20.75 ± 2.9 days (mean ± standard deviation). A steroid response occurred from tobramycin-dexamethasone in one case, prednisolone acetate in two cases, and PO prednisone plus difluprednate in our case (Table 1).

**Table 1:** Case Reports of IFS Occurring After SMILE BID: twice daily, DC: discontinue, gtts: eye drops, IFS: interface fluid syndrome, IV: intravenous, IOP: intraocular pressure, OD: right eye, OS: left eye, PO: oral, Q2h: every two hours, SMILE: small incision lenticule extraction

Study Group	Time After SMILE	Myopic Shift	Mechanism of Corneal Edema	Treatment
Zheng et al. [6]	24 days	-3.75 OD, -3.75 OS	IOP elevation after tobramycin-dexamethasone	DC steroids, IV mannitol, carteolol gtts BID, pranoprofen gtts QID, and 12.5 mg methazolamide BID
Moshirfar et al. [7]	21 days	-3.0 OD, -1.0 OS	IOP elevation from increased prednisolone Q1-2h	DC prednisolone, Combigan BID
Bansal et al. [8]	3 days	No refraction	Accumulation of tears in the interface from the torn edge of the cap	Hypertonic sodium chloride 5% eye drops Q2h
Trinh et al. [9]	21 days	-1.25 OD, -1.25 OS	IOP elevation after prednisolone	DC prednisolone
Our case	20 days	-4.25 OD, -2.75 OS	IOP elevation from difluprednate and PO prednisone	Brimonidine/timolol BID, bimatoprost daily, acetazolamide 500 mg PO BID

In all cases with a steroid response, stopping the offending agent was performed. Topical glaucoma drops were started in three out of four cases. Additionally, PO methazolamide or acetazolamide was started in two cases. Trinh et al. [9] had a complete resolution of IFS after stopping prednisolone acetate eye drops without adding topical or oral glaucoma medication, suggesting that IFS could solely be managed by stopping oral or topical corticosteroids.

Similar to SMILE, steroid-induced IOP elevation is the most common cause of early and late-onset IFS in LASIK [17-19]. Other less common causes of IOP elevation that can lead to IFS include failed glaucoma surgery, gas tamponade or silicone oil after vitreoretinal surgery, traumatic hyphema, retained viscoelastic after cataract surgery, and uveitis [20-23].

Endothelial dysfunction has also led to IFS in post-LASIK patients and may be a possible cause of IFS in post-SMILE patients as adoption increases among refractive surgeons. Fuchs endothelial dystrophy, phacoemulsification-induced endothelial decompensation, toxic anterior segment syndrome, cytomegalovirus endotheliitis, and Descemet membrane endothelial keratoplasty (DMEK) or Descemet stripping automated endothelial keratoplasty (DSAEK) graft failure have been possible causes of IFS in LASIK patients [24-27].

Although the diagnosis of IFS can be unclear with slit-lamp biomicroscopy, anterior segment optical coherence tomography (AS-OCT) or automated Scheimpflug densitometry can facilitate in confirming interface edema. AS-OCT can clearly demarcate fluid clefts within the potential space of the LASIK flap and cap after SMILE. The high resolution of AS-OCT can make visualization of the edema within the potential space of the LASIK flap or SMILE cap very obvious. Since IFS affects the transparency of the anterior corneal layer, automated Scheimpflug densitometry has been proposed as an objective tool to aid in diagnosis in post-SMILE patients [6]. Corneal densitometry increases with corneal edema, haze, or inflammation and can adequately identify interface edema [28]. Corneal densitometry can also accurately measure changes in corneal thickness, confirming progressive edema. In hindsight, automated Scheimpflug densitometry demonstrated significant interface edema with thickening on pachymetry in our case, which could have facilitated a correct diagnosis sooner. Among all cases of IFS after SMILE, 80% were diagnosed with either AS-OCT (2/5 cases) or automated Scheimpflug densitometry (2/5 cases), proving that both are useful tools in facilitating an accurate diagnosis.

The majority of cases of IFS in post-LASIK or SMILE patients are steroid-induced ocular hypertension. As a result, discontinuation of topical or oral steroids is crucial and may completely resolve the interface edema on its own [8]. Additional topical hypotensive medication may also be beneficial to acutely lower IOP. However, oral or IV hypotensive agents are typically not required.

## Conclusions

IFS, although uncommon, has been more widely documented in post-LASIK patients. Only a handful of cases have been documented in post-SMILE patients. As SMILE becomes more popular, postoperative IFS will likely become more common.

Among SMILE patients, IFS was most commonly associated with steroid-induced ocular hypertension and a myopic shift around 21 days postoperatively. A fluid cleft at the interface can be variable and may not be visible with SLE, confusing clinicians for DLK. Although elevated IOP is a prominent feature of most IFS cases, IOP measurements may be inaccurate secondary to the fluid interface, especially with GAT. Noncontact or handheld electronic tonometry are the preferred techniques for IOP calculation in these cases. However, AS-OCT or Scheimpflug densitometry may aid in quantifying interface edema needed to confirm a diagnosis when IOP is unclear. A corneal washout can immediately improve corneal edema, but the preferred treatment is discontinuing all steroid medication and starting glaucoma drops.
